# Basic FGF or VEGF gene therapy corrects insufficiency in the intrinsic healing capacity of tendons

**DOI:** 10.1038/srep20643

**Published:** 2016-02-11

**Authors:** Jin Bo Tang, Ya Fang Wu, Yi Cao, Chuan Hao Chen, You Lang Zhou, Bella Avanessian, Masaru Shimada, Xiao Tian Wang, Paul Y. Liu

**Affiliations:** 1 The Hand Surgery Research Center, Affiliated Hospital of Nantong University, Nantong, Jiangsu, China; 2 Department of Plastic Surgery, Rhode Island Hospital, The Alpert Medical School, Brown University, Providence, RI, USA; 3 Department of Molecular Biodefense Research, Yokohama City University, Yokohama, Japan; 4 Surgical Research and Gene Therapy Research, Department of Surgery, Roger Williams Medical Center, Boston University School of Medicine, Boston, MA, USA

## Abstract

Tendon injury during limb motion is common. Damaged tendons heal poorly and frequently undergo unpredictable ruptures or impaired motion due to insufficient innate healing capacity. By basic fibroblast growth factor (bFGF) or vascular endothelial growth factor (VEGF) gene therapy via adeno-associated viral type-2 (AAV2) vector to produce supernormal amount of bFGF or VEGF intrinsically in the tendon, we effectively corrected the insufficiency of the tendon healing capacity. This therapeutic approach (1) resulted in substantial amelioration of the low growth factor activity with significant increases in bFGF or VEGF from weeks 4 to 6 in the treated tendons (p < 0.05 or p < 0.01), (2) significantly promoted production of type I collagen and other extracellular molecules (p < 0.01) and accelerated cellular proliferation, and (3) significantly increased tendon strength by 68–91% from week 2 after AAV2-bFGF treatment and by 82–210% from week 3 after AAV2-VEGF compared with that of the controls (p < 0.05 or p < 0.01). Moreover, the transgene expression dissipated after healing was complete. These findings show that the gene transfers provide an optimistic solution to the insufficiencies of the intrinsic healing capacity of the tendon and offers an effective therapeutic possibility for patients with tendon disunion.

Tendon injuries constitute one of the most common disorders of the human body, affecting 1 in 2,000 people each year, with the tendon injuries to the hand and wrist occurring in 1 in 2,700 people each year^1^,^2^. These tendon injuries can result from trauma, overuse, or age-related degeneration from work, daily life, and sports activities. Injuries to tendons, tendon-bone-junctions, and related tissues (such as ligaments) can occur in numerous areas of the body. People with such injuries constitute a large proportion of the patients treated in emergency rooms, inpatient surgical departments, outpatient clinics, and rehabilitation facilities. Damaged tendons heal poorly; their surgical repair frequently ends in unpredictable rupture or impaired extremity motion due to insufficient healing capacity. The treatment of damaged tendons remains a challenge in medicine because of the insufficiency of the healing capacity of the tendon itself and lack of method to increase the biological healing strength.

Tendons, particularly those covered by an intrasynovial sheath, have very limited vascular supply, lack sufficient cellularity, and have low growth factor activity. These structural or biological features account for the weak healing strength of tendons after injury^3^,^4^,^5^. So far, treatment options for injured tendons have not proven adequate to correct the insufficiency of intrinsic healing capacity of intrasynovial tendons^4^,^5^,^6^,^7^,^8^,^9^,^10^,^11^,^12^,^13^,^14^,^15^, despite preliminary findings indicating better healing responses of extrasynovial tendons to some therapies in animal models^16^,^17^,^18^. We aimed at developing a new therapeutic approach that corrects the fundamental problem underlying intrasynovial tendon healing with introduction of select growth factor genes to the tendon producing supernormal amounts of these factors required during the early tendon healing period.

We tested efficiency of the transfer of a number of growth factor genes in promoting tendon healing *in vitro* and *in vivo* and found basic fibroblast growth factor (bFGF) and vascular endothelial growth factor (VEGF) are among the most potent stimulators of tenocytes (tendon fibroblasts) proliferation and type I collagen production. An adeno-associated viral (AAV) vector was the gene delivery vehicle in our study because this virus is non-pathogenic. We hypothesized that transfer of bFGF or VEGF genes using AAV type 2 (AAV2) vectors would augment productions of growth factors, collagens, and their modulators in the treated tendons, that eventually significantly enhance the healing strength over a critical period of the tendon healing. We expected that the bFGF or VEGF gene therapy corrects the insufficiency of the intrinsic healing capacity, leads to quicker and more robust tendon healing after surgery, and may become an efficient biological treatment modality for the patients with injured tendons.

## Results

### Experimental plan

We completely severed chicken flexor tendons, i.e., floxor digitorum profundus (FDP) tendons and injected AAV2 vectors carrying transgenes (bFGF or VEGF genes) or sham AAV2 vectors immediately before repairing the tendon surgically. The vectors were introduced to the tendon through micro-injection to both tendon stumps through cross-sections of the tendon cut. We used non-injected tendons as non-treatment controls.

We injected a single dose of AAV2-bFGF or AAV2-VEGF (2 × 10^9^ viral particles/tendon) into transversely lacerated digital flexor tendons of chickens. The dose of injection was decided according to a pilot study using the same chicken tendon injury and repair model. In the pilot study, we injected 2 × 10^7^, 2 × 10^8^, 2 × 10^9^, and 2 × 10^10^ viral particles (vp) to each tendon and found an increase in healing strength by 30–40% when the amount of vectors increased from 2 × 10^7^ to 2 × 10^8^ vp or greater, but no statistical difference was found between tendons injected with 2 × 10^8^, 2 × 10^9^ or 2 × 10^10^ vp (8 tendons at each dose, statistical power > 0.80).

### bFGF or VEGF gene delivery prevents the drop of bFGF or increases VEGF gene expression in healing tendons

We harvested tendons injected with AAV2-bFGF or AAV2-VEGF, or sham AAV2 vector, and the tendons in non-injection controls over a 16-week period at 8 time-points (weeks 1, 2, 3, 4, 6, 8, 12, and 16), covering the early, middle, and late tendon healing stages. Real-time polymerase chain reactions (qPCR) and western blot were performed to analyze expression of transferred bFGF or VEGF genes, respectively.

**Figure 1:**
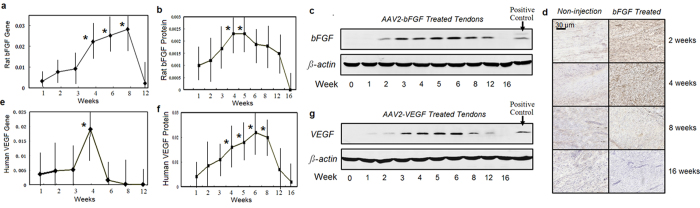
Transgene and protein production in AAV2-bFGF or AAV2-VEGF injected tendons. (**a**) Transgene (rat bFGF) expression in AAV2-bFGF injected tendon increased from weeks 1 to 3, peaked from weeks 4 to 8, dropped drastically after week 8, and was very low at week 12. *indicate the data significantly greater than that at other time-points (p < 0.05 or p < 0.01). (**b**) bFGF protein levels relative to beta-actin. *indicates the data significantly greater than that at weeks 1, 2, 12, 16 (p < 0.01 or p < 0.01).(**c**) Representative pictures of gel electrophoresis of western blot using mouse-anti-rat bFGF antibody. Rat bFGF was increased from weeks 2 to 4, peaked at weeks 4 and 5, and declined at weeks 6 to 12. The bFGF was not detectable at week 16. (**d**) immunohistochemistry showing the changes of the bFGF (chicken and rat origins) in the AAV2-bFGF injected and non-injection control tendons upto week 16. The bFGF was increased at weeks 2 and 4 in the AAV2-bFGF injected tendon. (**e**) Transgene (human VEGF) expression in the AAV2-VEGF injected tendon. Transgene expression peaked at week 4. The expression was minimal at week 6, 8, and 12. *indicates the data significantly greater than that at other time-points (p < 0.05 or p < 0.001). (**f**) Western blot analysis showed gradual increase in the expression of human VEGF from weeks 1 to 6. The VEGF peaked at week 6 and dropped thereafter. Expression of the VEGF relative to beta-actin is shown. *indicates the data significantly greater than that at week 1, 12, or 16 (p < 0.01 or p < 0.001). (**g**) gel pictures showing the changes in human VEGF. The VEGF was not present in the gel at week 16. The sample number (n) was 6 for analysis of gene expression and 4 for western blot analysis at each time point in each group.

The bFGF gene delivered to the chickens was of rat origin, while the VEGF was of human origin. By designing primers that specifically amplify rat bFGF segments using qPCR, we were able to assess the changes in the expression levels of the exogenous bFGF gene from post-surgical weeks 1 to 16 (Fig. 1a). The expression of bFGF transgene was detected at week 1, and gradually increased from weeks 2 to 8, then dropped from weeks 8 to 12. The bFGF transgene expression was statistically greater at weeks 4, 6, and 8 than that at 1, 2, and 12 (p < 0.05 or p < 0.001). Expression of the bFGF transgene became undetectable at week 16. At 1 to 4 weeks, the expression of the endogenous chicken bFGF was increased significantly in the tendon treated with AAV2-bFGF compared with that in those treated with sham vectors or in non-injection controls (p < 0.05 or p < 0.01). In the non-injection control tendons, the expression of the endogenous bFGF decreased significantly at weeks 1 to 5 after injury compared with healthy tendons (p < 0.05 or p < 0.01).

Western blot analysis using mouse-anti-rat bFGF antibody showed similar increases in the transgene in weeks 2 and 3, a peak from weeks 4 to 8, and no detectable exogenous bFGF at week 16 (Fig. 1b,c). Immunohistochemical staining verified an increase in the total amount of bFGF of the AAV2-bFGF treated tendons (Fig. 1d).

Similarly, VEGF transgene of human origin was detectable at weeks 1 through 8 and peaked at week 4, and the VEGF transgene expression was statistically the greatest at week 4 (p < 0.05)(Fig. 1e). At weeks 2, 3, and 4, the expression of the endogenous VEGF in the tendon treated with AAV2-VEGF was increased significantly compared with that in the tendons injected with sham vectors or in non-injection controls (p < 0.01). From weeks 2 to 12, the human VEGF was detected in the tendon by western blot using mouse-anti-human antibody, with peak in weeks 4 to 8 (Fig. 1f,g). Production of the exogenous VEGF was not detectable at week 16 (Fig. 1g).

We used a set of primers to amplify a segment of the bFGF gene identical in chicken and rat bFGF genes. We found that levels of compound expression of both endogenous and the transferred bFGF genes increased in the early and middle healing periods (weeks 2 to 8) and the chicken bFGF gene was upregulated in this period in the AAV2-bFGF injected tendons. These increases were in contrast to the non-injection controls, which demonstrated down-regulation of their bFGF gene expression after injury, with the levels remaining low until week 8. From weeks 2 to 8, we also found significant increases in the expression of VEGF genes in the AAV2-VEGF injected tendon using primers amplifying a segment of gene common to both chicken and human VEGF genes.

### bFGF and VEGF gene delivery produces an early increases in type I collagen production and modulates type III collagen production and other extracellular matrix gene expression

**Figure 2:**
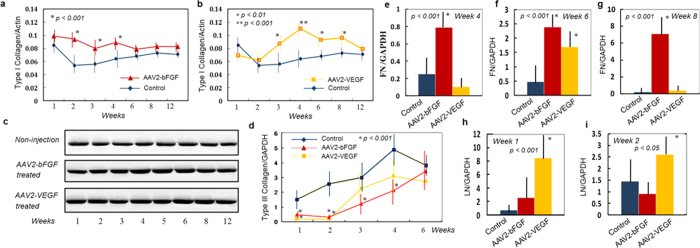
Changes in expression of extracellular matrix after AAV2-bFGF or AAV2-VEGF injection to the tendons. (**a**) Type I collagen were significantly increased at weeks 2, 3, and 4 in the AAV2-bFGF injected tendon compared with the non-injection controls (p < 0.001). (**b**) Type I collagen was significantly increased at weeks 3, 4, 6, and 8 in the AAV2-VEGF injected tendon (p < 0.01, or p < 0.001). (**c**) gel pictures showing the changes in protein levels of type I collagen. Note an earlier increase (weeks 2 to 5) of the collagen I after AAV2-bFGF injection, but a greater and more persistent increase (up to week 8) after AAV2-VEGF injection. (**d**) Changes in type III collagen gene expression of the AAV2-bFGF and AAV2-VEGF injected tendons compared with non-injection controls (p < 0.001, 1 to 4 weeks after AAV2-bFGF treatment, and 1 and 2 weeks after AAV2-VEGF treatment). (**e–i**) showing the real-time PCR analysis of changes in expression of the fibronectin (FN) at weeks 4, 6, and 8 and the laminin (LN) at weeks 1 and 2. Statistical significance is shown in the graph. * indicates the data of significant difference from those in the non-injection controls. Sample sizes at each time point in each group were 6 to 8 for gene expression analysis and 5 or 6 for western blot analysis.

The main determinant of a successful tendon repair is the early gain of mechanical strength, which depends on robust synthesis of collagens and other extracellular matrix components to bridge the repair site. Type I collagen is particularly important for the gain of healing strength. Presence of the type III collagen early in repaired tendon is less favorable as it does not contribute much to the tensile strength of an intact or healing tendon. A primary goal of augmenting tendon strength should be to increase type I collagen and decrease type III collagen. Western blot analysis showed significant increases in expression of type I collagen in the AAV2-bFGF or AAV2-VEGF treated tendons (Fig. 2a–c), with significant increases at weeks 2, 3, and 4 in AAV2-bFGF treated tendons (Fig. 2a) and at weeks 3, 4, 6, and 8 in AAV2-VEGF treated tendons (Fig. 2b)(p < 0.01 or p < 0.001). The amount of type I collagen was not increased significantly at week 1 or 12 after either therapy. After either therapy, type III collagen gene expression was dramatically down-regulated in the early weeks after surgery, i.e., week 1 and 2 (p < 0.05 or p < 0.01). In addition, down-regulation of type III collagen persisted at week 3 and 4 after AAV2-bFGF treatment (Fig. 2d). Expression of the type I and III collagen genes was very low in normal tendons, being 0.22 and 0.07 relative to the glyceraldehyde-3-phosphate dehydrogenase (GAPDH) gene, respectively. Both genes had significantly lower levels of expression in normal tendons than that in the surgically repaired tendons (p < 0.001).

We used qPCR to examine expression of aggrecan (AGC), decorin (DCN), fibronectin (FN), laminin (LN), and fibromodulin (FMOD) genes at postoperative weeks 1, 2, 3, 4, 5, 6, and 8. We identified that increases in gene expression of FN at weeks 4, 6, and 8 after AAV2-bFGF treatment (Fig. 2e–g) and at week 6 after AAV2-VEGF treatment (Fig. 2f); similar increases were found of LN at weeks 1 and 2 after AAV2-VEGF treatment (Fig. 2h,i). Expression of AGC, DCN, and FMOD genes was not significantly changed by the gene therapy.

### bFGF and VEGF gene delivery modulates metabolism of the tendon to favor healing

The metabolism of the extracellular matrix affects collagen production and degradation. Therefore, we determined gene expression and protein production of several principle regulators of metabolism. We assessed the expression of matrix metalloproteinases (MMPs)(MMP1, 2, 3, and13) and tissue inhibitors of metalloproteinases (TIMPs) (TIMP2 and 3) using qPCR and western blot.

**Figure 3:**
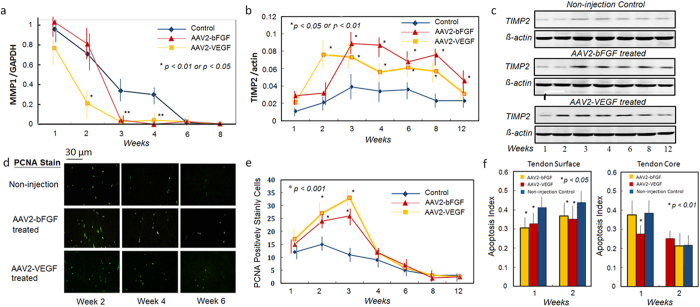
Changes in regulators MMPs and TIMPs of metabolism in the AAV2-bFGF and AAV2-VEGF treated tendons. (**a**,**b**) significant changes in the expression of the MMP1 and TIMP2 was found in the tendons after either AAV2-bFGF or AAV2-VEGF treatment (n = 6, in each group at each time point), typically from weeks 2 to 8 (*p < 0.05 or p < 0.01, compared with non-injection controls). (**c**) western blot gel pictures show that the TIMP2 was activated after the therapy from weeks 2 to 8 to inhibit collagen degradation. (**d,e**) PCNA staining showed significant increases in the positively-stained cells after injection of AAV2-bFGF or AAV2-VEGF at weeks 2 and 3. (**d**) the representative pictures (200 X magnification). (**e**). Data presented are from 6 fields of each of 6 tendon samples per group under 200 X magnification. *indicates data of significant difference from the non-injection controls at weeks 2 and 3.(**f**) apoptosis index of the AAV2-bFGF or AAV2-VEGF injected tendons and non-injection controls at weeks 1 and 2 (n = 6, each group at each time point, *p < 0.05 or p < 0.01). No significant difference was found in the number of the PCNA positively stained cells and apoptosis index in these groups at weeks 4, 6, 8, and 12 (data not shown). *indicates the data of significant difference from the non-injection controls. The data of sham vector controls (not shown) were not significantly different from the non-injection controls.

We found significant down-regulation of the MMP1 gene at weeks 3 and 4 in AAV2-bFGF treated tendons, and at weeks 2, 3, and 4 in the AAV2-VEGF treated tendons as compared with non-treated controls (p < 0.01) (Fig. 3a). Expression of the MMP1 gene was 0.9 ± 0.2 (relative to GAPDH) in normal tendons, which was not significantly different from that in the injured tendon at weeks 1 and 2. We found significant down-regulation of the MMP3 gene at week 4 in AAV2-bFGF treated tendons (p < 0.01), and from weeks 1 to 4 in AAV2-VEGF treated tendons (p < 0.05 or p < 0.01). In contrast, TIMP2 gene expression was up-regulated at weeks 3 to 12 after AAV2-bFGF treatment, and at weeks 2 to 8 after AAV2-VEGF treatment (Fig. 3b,c). Expression of the TIMP2 gene was 0.01 ± 0.01(relative to GAPDH) in normal tendons, which was not significantly different from in the injured tendon at week 1. TIMP3 gene expression was up-regulated only transiently at weeks 1 and 2 after AAV2-bFGF treatment and at week 4 after AAV2-VEGF treatment.

### bFGF or VEGF gene delivery increases proliferation and prohibits apoptosis of tendon fibroblasts

We quantified the proliferation of tenocytes using proliferating cellular nuclear antigen (PCNA) staining. PCNA positive cells were found to be increased significantly at weeks 2 and 3 after either AAV2-bFGF or AAV2-VEGF treatment (Fig. 3d,e). We also examined apoptotic cells of the tendon surface and core regions. The apoptosis index (AI) dropped significantly at weeks 1 and 2 after AAV2-bFGF or AAV2-VEGF treatment on the tendon surface and at week 1 after AAV2-VEGF treatment in the core (Fig. 3f).

### bFGF or VEGF gene delivery enhances the healing strength in the critical healing period

**Figure 4:**
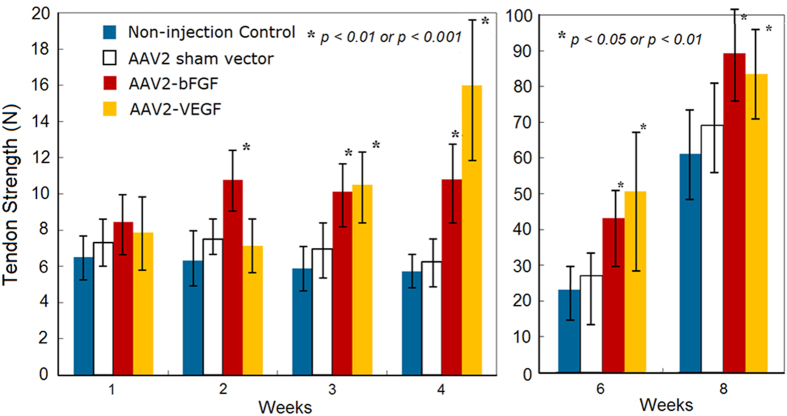
Tendon healing strengths (data of weeks 1, 2, 3, 4, 6, and 8 shown, n = 12, each group at each time point). Compared with non-injection and sham vector controls, the strengths of the AAV2-bFGF injected tendon had significant increases from week 2 and lasted up to week 8 (p < 0.01 or p < 0.001). In contrast, AAV2-VEGF treatment brought more robust and significant increases at week 3 (p < 0.01) and week 4 (p < 0.001). The strengths of the tendons injected with AAV2-VEGF were significantly greater compared with non-injection controls or sham vector injection controls at weeks 6 and 8 (p < 0.05 or p < 0.01). No significant difference in the strengths between the sham vector and non-treatment controls (p > 0.05, statistical power > 0.80). Compared with the strengths of non-injection controls, the percent increases in the strength were 72%, 68% and 91% for the AAV2-bFGF treated tendons at weeks 2, 3, and 4, respectively, and the increases were 82% and 210% for the AAV2-VEGF treated tendons at week 3 and 4, respectively. *indicates the data of significant difference from those in the non-injection and sham vector controls at individual time points.

Using an Instron tensile testing machine (Model 4411, Instron Inc., Norwood, Mass.), we measured the healing strength of the tendons injected with AAV2-bFGF or AAV2-VEGF at postoperative day 0, and at weeks 1, 2, 3, 4, 6, and 8. The healing strength is the most important mechanical parameter of actual effects of interventions on tendon healing. The gain in the strength is the ultimate goal of therapy. From weeks 1 to 4, the non-injection or sham vector control tendons typically exhibited “no-gain” in strength. By contrast, earlier increases in strength were recorded after either AAV2-bFGF or AAV2-VEGF treatment. Notably, healing strength after AAV2-bFGF was significantly increased at weeks 2, 3, 4, 6, and 8 compared with non-injection or sham vector injection controls (Fig. 4). After AAV2-VEGF injection, the strength of the tendons was significantly increased starting at week 3 and continually up to week 8. The increases in strength were dramatic—an increase by 68–91% in the AAV2-bFGF treated tendon, and an even greater increase in the AAV2-VEGF treated tendon—by 82–210%. In comparing the effectiveness of AAV2-bFGF with that of AAV2-VEGF, we found earlier significant effects after AAV2-bFGF treatment; however, the degree of increased strength of AAV2-VEGF injected tendons was greater than that of AAV2-bFGF injection at week 4 and 6 (Fig. 4). Injection of sham vectors did not significantly change strength compared to tendons in non-treatment controls (p > 0.05, statistical power > 0.80). At the end of week 8, the strengths of the tendons treated with either AAV2-bFGF or AAV2-VEGF were not statistically different from those of healthy tendons (p > 0.05, statistical power > 0.80). Twelve healthy FDP tendons of the chickens were tested; the ultimate strengths were 91 ± 14 N.

**Figure 5:**
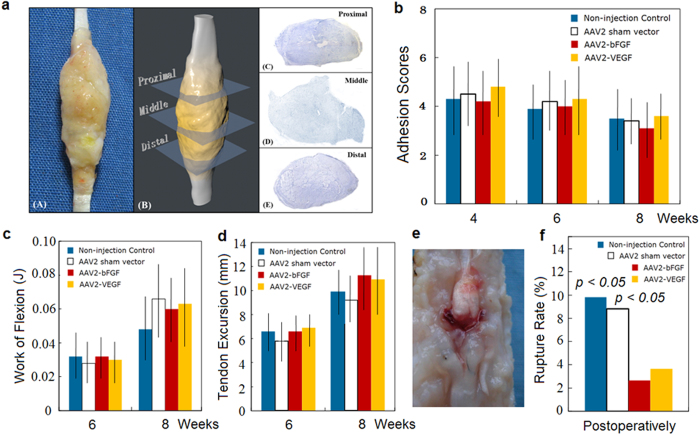
Effects of AAV2-bFGF and AAV2-VEGF injection to the tendon on adhesion formation and amplitude of tendon movement. (**a**) a three-dimensional analysis method for quantification of adhesions around the tendon. The tendon is sectioned through 3 cross-sectional levels (0.5 cm apart, with the middle section at the site of tendon repair) and is stained histologically. The area of adhesions and the ratio of adhesions to the healing tendons are computed to obtain adhesion scores. (**b**) adhesion scores (n = 8, each group at each time point) are presented. No significant difference was found in the scores (shown in **b**) and area of adhesions (not shown). (**c**) work of flexion of the toes (n = 12, each group at each time point). (**d**) tendon excursions under 10 N load to the repaired FDP tendon (n = 12, each group at each time point). No significant differences were found in the work of flexion and tendon movement at week 6 and 8 (p > 0.05, statistical power > 0.85). (**e**) a picture shows a typical tendon rupture. (**f**) overall rate of tendon ruptures recorded during dissection in the samples for mechanical test at weeks 4, 5, 6, and 8 (48 toes at each group) after surgery. Significant differences in the rupture rate were noted between the AAV2-bFGF or AAV2-VEGF injection, sham vector and non-injection groups. P values shown are comparison of the non-injection and sham vector groups with the AAV2-bFGF or AAV2-VEGF injection groups.

No significant increases in amount of adhesions and in work needed to flex the repaired toes were not found in the treatment groups (p > 0.05, statistical power > 0.85)(Fig. 5a–d). The overall rupture rate of repaired tendons was significantly greater in both control groups than in treatment groups (p < 0.01)(Fig. 5e,f).

### Production of supranormal amounts of bFGF or VEGF ceases after healing is complete

We measured rat bFGF or human VEGF in the tendon upto16 weeks (Fig. 1c,g); at that point, tendon healing is complete. Both gene expression and amount of bFGF or VEGF protein present in the treated tendons decreased from weeks 12 to 16 to minimal or undetectable levels (Fig. 1a–c,e,f). At week 16, levels of bFGF and VEGF in the treated tendons returned to the levels in non-injection controls (Fig. 1d).

### Tendon structures in histology

**Figure 6:**
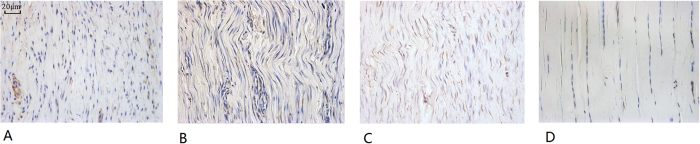
Three sections of healing tendons at week 6 and the uninjured tendons shown. (**A**) AAV2-bFGF treated tendon, (**B**) AAV2-VEGF treated tendon, (**C**) non-injection control tendon, and (**D**) uninjured tendon. Morphologically, the cellularity and collagen formation in AAV2-bFGF or AAV2-VEGF treated tendon (**A,B**) are greater than those in the non-treatment control (**C**) or uninjured tendon (**D**). This is at the beginning of the tendon remodeling, so cellularity in the tendon still much more robust in these healing tendons. The sections stained for immunohistochemistry were used for the observation (X400, magnification). Section shown in (**A,C,D**) was stained with mouse anti-rat bFGF antibody (05–118, Millipore Corp., Billerica, Mass.) and that shown in b was stained with mouse anti-human VEGF (Santa Cruz, Dallas, Texas).

At week 8 and later, we observed that the histological sections show better structural remodeling with more regularly aligned collagens in the treated tendons compared with sham vectors and in non-injection controls. However, the structures were still not normal even at week 12, which indicates that structural remodeling took more than 8 or 12 weeks. At week 6, the cellularity in the treatment tendons is still more prominent than that in the non-injection controls, and the collagens appeared to be more robust in the treatment groups (Fig. 6A–D).

## Discussion

Intrasynovial tendons structurally and metabolically resemble articular cartilage^3^,^4^. Intrasynovial tendons are largely devoid of vasculature, healing of injured areas is poor, and participation of adhesions in the tendon healing is common^3^,^4^,^19^,^20^,^21^. Clinically, the repaired tendons need to move during early healing to restore its gliding surface. If the tendon is not moved after surgical repair, adhesions arise, that jeopardizes tendon motion. However early motion carries the risk of disrupting the repair^22^,^23^. The central tenet underlying these problems is the lack of sufficient healing capacity of the tendon intrinsically. To our knowledge, almost all efforts through biological approaches have failed to enhance healing strength of intrasynovial tendons dramatically and at multiple time-points, though some produced marginal gain of strength at a single postsurgical time-point by means of a coated suture or other methods^9^,^10^,^11^,^12^,^13^,^14^,^15^. Failure in such attempts was highlighted by use of controlled-release system loaded with bFGF, which stimulated biological responses, but was unable to increase mechanical strength^13^,^14^.

Our investigation was based on a series of prior studies in which we identified the growth factors likely relevant or critical to tendon healing, developed gene delivery methods through micro-injection to the tendon, and tested different dosages to maximize gain in mechanical strength. The bFGF gene was chosen for a number of reasons: First, bFGF is one of best-characterized growth factors for tissue repair, and its major action is promotion of collagen production, tendon development, and proliferation of tenocytes^24^,^25^,^26^. Second, bFGF plays a critical role in differentiation of tendon tissues^27^,^28^. Third, based on our previous data, bFGF is down-regulated during tendon repair^29^. Therefore, low levels of bFGF may be a principle reason for poor healing potential of the tendon. Fourth, introduction of bFGF gene to the tendon could promote expression of a series of growth factor genes as shown in our previous studies^29^. AAV2-VEGF was selected based on the fact that vascular supply to the tendon is poor, and an assumption that VEGF may be particularly needed in the healing process of the tendon. In our previous study, we detected low levels of VEGF expression in the healing tendon^29^. This observation led us to believe that low VEGF activity may be partly responsible for the slow or weak healing responses in the injured tendons. The use of serotype 2 of the AAV vectors was based on our prior experiments comparing transfection efficiency of different serotypes of this vector^30^. The major basis of our selection is a pilot study of expression profile of different growth factors in the early stages of tendon healing, in which we found that bFGF gene expression is minimal and downregulated in the first 3 weeks, and that VEGF gene expression is minimal after tendon injury^30^. Following the pilot study, we proved that transfers of bFGF or VEGF genes to the tenocytes enhance of expression of the type I collagen gene in a tenocyte culture setting^31^,^32^,^33^,^34^,^35^. The reasons of not transferring transforming growth factor (TGF)- β1gene to the tendon are that this gene is highly expressed in the healing tendon and that TGF-β1 is responsible for restrictive scar around the tendon decreasing gliding. We did not consider transferring epithelial growth factor (EGF) or connective tissue growth factor (CTGF) genes, because bFGF and EGF have similar function, but bFGF likely acts on fibroblasts (such as tenocytes) efficiently, and CTGF and TGF-β1 share similar function^34^,^35^.

In this study, delivery of either bFGE or VEGF genes through AAV2 vectors improved the tendon strength in the early and middle healing stages. Notably, this gain of strength is achieved without the cost of increase in associated adhesion formation or resistance to tendon gliding. This therapy offers a highly efficient way of improving tendon strength. The impact of this therapeutic approach is impressive in our animal model, producing an increase in strength by 68 to 210%, which is likely ample to prevent tendon gapping or disunion of the tendons. This study illustrates a way through which intrinsic healing capacity is enhanced and the “no-gain” period of tendon strength recovery in the initial a few weeks after repair can be converted to a steady gain in the period when the tendon frequently disrupts.

No increase was found in resistance to tendon motion or severity of adhesion in the tendon treated with AAV2-bFGF or AAV2-VEGF as compared with non-treatment and sham vector controls. Notably, expression of type III collagen was down-regulated from weeks 1 to 4 after AAV2-bFGF treatment and at week 1 to 2 after AAV2-VEGF treatment (Fig. 2d); thereafter the type III collagen expression increased to the level identical to that of the non-injection controls. The increase in type III collagen at week 6 would not increase the amount of adhesions, because adhesions form around the tendon form during the first weeks of the healing tendon. In the later healing, adhesions do not increase but rather remodel to allow greater tendon gliding. Down-regulating type III collagen in the first a few weeks after surgery lead to deposition of a greater amount of mature collagen (type I collagen), favoring earlier gain in the strength.

Our findings regarding changes in the extracellular matrix (and its regulators) provide additional mechanistic explanation for gain in the strength of the treated tendons. With AAV2-bFGF and AAV2-VEGF treatment, MMPs were down-regulated and TIMPs were up-regulated; both of these changes act to slow down degradation of extracellular matrix. In addition, the increases in proliferation rate of tendon fibroblasts were paired with inhibition of cell apoptosis. The mechanism of these therapies, therefore, is likely an initial increase in tendon cell proliferation paired with inhibition of cellular apoptosis, followed by supernormal production of type I collagen with inhibition of type III collagen, and an overall slow-down of collagen degradation as a result of changes in activities of MMPs and TIMPs. Our findings suggest that these molecular events effectively transform a lengthy inactive early-to-middle healing period to a biologically robust healing period, leading to impressive gain of strength.

We are aware that Thomopoulos *et al*.^13^. found significant increases in vascularity, cellularity, type III collagen, and adhesion formation in the tendons after bFGF being delivered through a controlled release system. Tendon healing strength was not increased as compared to the tendons that received operative repair alone^13^. In contrast, after AAV-bFGF treatment, we did not find increases in vascularity, type III collagen, and adhesions. Our assumption for molecular basis of the differences is that the AAV2 vector ensures a gradual increase in production of the bFGF in the first a few weeks in the healing tendon, but the amount of bFGF protein released from a controlled release system decreases progressively after surgery. We did not find angiogenic effects of AAV2-bFGF over the tendon surface in histological sections. However we noted mild inflammatory changes over tendon surface in the first two weeks after AAV2-bFGF and AAV2-VEGF injection. We are not sure whether the inflammation was caused by the AAV2 vector or increases in bFGF in the tendon. Previous investigations documented that AAV2 causes transient inflammation in the epitenon 2 to 3 weeks after injection^36^. In a previous study^29^, we recorded upregulation of multiple growth factors after AAV2-bFGF treatment. The impact of activation of multiple growth factors may be multifaceted, including eliciting mild inflammatory changes and producing an even more robust downstream effect that strengthens the healing tendon.

Aiming at conducting a comprehensive pre-clinical study, in this study we set out to assess the effects of AAV2-bFGF and AAV2-VEGF treatment upto 16 weeks after surgery. We verified that expression of transgenes peaked during the early and middle postoperative periods, and declined to minimal or even undetectable levels after week 12. The tendon healing typically takes up to 8 to 12 weeks, though structural remodeling may take long later^4^,^5^. Most attractive of this tested gene therapy approach is that, neither effects of transgene expression of this approach nor transgene itself were detectable 12 weeks later. The observations confirm our hypothesis that this approach introduces genes to healing tendons, increases the transgene expression in the healing process of the tendon, and then ceases production of the transgenes once its “work” has been accomplished. According to the work of others^37^,^38^,^39^,^40^, such a timely switch off of transgene expression introduced by the AAV2 vector may relate to an immune reaction to the transgene products. An alternative possibility is gene silencing or cell turnover^39^,^40^,^41^,^42^,^43^. Long-term or constitutive expression of transgenes is a much sought-after feature of gene therapy targeting congenital diseases or tumors^41^,^42^. However, for tissue healing, a transient supranormal production of growth factors is the only goal. Termination of transgene expression after healing is much desirable for the gene therapy application to traumatized tissues. Whether expression of transgenes will be switched off in patients would need careful evaluation when this treatment is moved to clinical trial in future, because immune responses is absent if human VEGF gene is introduced to the patients. A few recent studies highlighted non-viral or physical approaches to introduce genes to the Achilles tendon, an extrasynovial tendon^44^,^45^. Our group also used nanoparticles to deliver genes to tendons to inhibit adhesion formation^46^. These approaches appear just as efficient in delivering genes to the healing tendons through AAV2 vectors. However, none of above reports have targeted to increase the healing strength of intrasynovial tendon. It is unknown whether the tendon healing strength can be increased significantly at multiple time points through these gene delivery methods^43^,^44^,^45^,^46^.

We recorded distinct patterns of tendon healing strength gain between two growth factor gene therapies. The AAV2-bFGF brought about an earlier gain in the strength (starting as early as from week 2) compared with AAV2-VEGF (starting from week 3); but the amplitude of strength gain was greater after AAV2-VEGF treatment. We are unable to fully explain why the two vectors led to different patterns of strength gain. One assumption is that the bFGF, a potent stimulator of collagen production, has a direct and immediate impact on the production and metabolism of collagens. Actions of VEGF might be delayed, but more potent, in the tissue largely devoid of vascularity. In other tissues, it was reported that VEGF enhanced collagen production through MAP kinase signal pathways^47^. The action of VEGF may relate to its diverse signaling pathways, which varies according to matrix environment of tissues^47^. We found that expression of type I collagen, fibronectin, and laminin increased significantly after gene therapy, without significant increase in fibromodulin, aggrecan and decorin. We do not know why the gene therapies acted differently on expression of these molecules, and why molecules such as fibromodulin (responsible for collagen assembly) did not increase in the treated tendons. However, our findings likely indicate that a mechanism for the increase in mechanical strengths of the treated tendons is slowing the degradation of collagen fibers (including those in the tendon ends before injury and those newly formed during healing), as supported by changes in MMPs and TIMPs, and type I collagen production.

This preclinical animal experiment holds great promise for treating tendon injury. The micro-injection of AAV vectors to the tendons is simple, yet effective. The AAV vectors have been used in a number of animal studies and clinical trials, and thus far, have been safe^33^,^38^,^48^,^49^,^50^,^51^,^52^,^53^,^54^,^55^,^56^. We verified that these vectors were not expressed in vital organs (i.e., brain, heart, lung, liver, ovary, etc.). Although our data indicate similar treatment efficacy profiles, we noted slightly different effects between AAV2-bFGF and AAV2-VEGF. Our mechanical tests showed that neither therapy potentiates adhesion formation around healing tendons. All these findings make both studied therapies appropriate candidates for clinical trials. We anticipate, with modification of vector construction, both therapeutic approaches will be appropriate for clinical trials and hold great promise of correcting weak healing potential of the intrasynovial tendon to combat different problems associated with tendon repair in the clinical arena.

## Methods

### Chicken tendon injury, surgical repair model and group division

The animal experimentation was conducted in accordance with the approved guidelines of Nantong University and National Experimental Animal Regulation. This study was approved by the Experimental Animal Care Committee of Nantong University.

#### Animals

Adult white Leghorn chickens were used as experimental models, because the flexor tendons in chicken toes are similar to those in human digits and are often used for investigation of digital flexor tendon surgery^57^,^58^,^59^. Among 263 chickens were used for this study, 12 chickens were used for obtaining data of strengths in healthy tendons and 53 chickens for testing strength of the tendons immediately after surgical repair, or obtaining molecular and histological data in healthy tendons or tendon with only surgical repair. 198 chickens (396 long toes of both feet) were used for mechanical tests and/or harvesting tendon samples for analysis of gene expression, western blot analysis of proteins, or histological examination.

#### Surgical Procedures and Groups

The long toes of chickens were randomly assigned to 4 experimental arms according to differing treatments administered at surgery. The chickens were anesthetized by intramuscular injection with ketamine (50 mg/kg of body weight). The toes were operated under sterile conditions and tourniquet control using elastic bandages. A zigzag incision was made in the plantar skin between the proximal interphalangeal (PIP) and distal interphalangeal (DIP) joints, which is equivalent to zone 2 in the human hand^60^,^61^. Through a 1.0-cm long longitudinal incision through the tendon sheath, a transverse cut of the FDP tendon was made with a sharp scalpel at the level about 1.0 cm distal to the PIP joint with the toe in extension. The long toes were divided as follows:

#### Group 1. Non-treatment control

Tendons did not receive any injection. *Group 2. Sham-vector treatment control:* 2 × 10^9^ vp of AAV2 sham vector diluted in 20 μl of physilogical saline were injected into each tendon. *Group 3. AAV2-bFGF injection group:* 2 × 10^9^ vp of AAV2-bFGF in 20 μl of physilogical saline were injected into each tendon. *Group 4. AAV2-VEGF injection group:* 2 × 10^9^ vp of AAV2-VEGF in 20 μl of physilogical saline were injected.

The cut tendon was repaired with the modified Kessler method with 5-0 sutures (Ethilon; Ethicon, Somerville, New Jersey). The incised sheath was left open and the skin was closed with interrupted sutures. The operated toes were immobilized in a dressing wrap with adhesive tape in a semiflexed position after surgery.

A micro-injection needle was used for vector injection through the lacerated tendon cross-sectional surface at the depth of 0.5 cm at four sites (2 sites in either tendon stump). 5 × 10^8^ particles of AAV2-bFGF vector were injected to each of four sites in the stumps of the cut tendon ends before repair, yielding a total injected dose of 2 × 10^9^ in each tendon.

The operated toes were divided into subgroups according to the timing of harvest at postoperative day 0, and weeks 1, 2, 3, 4, 5, 6, 8, 12, and 16.

### Gene Transfer Units—AAV2-bFGF and AAV2-VEGF—Vector Construction and Production

Single-stranded AAV2 vectors were used. The AAV2-bFGF vector plasmid was constructed as we described in previous publications^34^,^35^. The bFGF gene is of rat origin (Gene bank accession no. X07285). The AAV2-VEGF vector plasmid pAAV2-VEGF was constructed by inserting human VEGF gene (Gene bank accession no. AF486837) encoding human VEGF 165 isoform into pAAV-MCS (Stratagene, La Jolla, Calif.) The AAV2 sham vector plasmid was purchased from Stratagene. AAV2-bFGF, AAV2-VEGF and sham vector were subsequently produced and purified in Vector BioLabs (Philadelphia, Penn.).

### Real-time PCR

Total RNA was isolated and was reversely transcripted to complementary DNA (cDNA). Expression of genes was analyzed by real-time quantitative polymerase chain reactions (qPCR) using the Eppendorf Mastercycler ep realplex (2S; Eppendorf, Hamburg, Germany). Expression of the transcriptions was normalized to the GAPDH gene to standardize comparison.

### Immunohistochemistry and immunofluorescence

After harvest, the tendons underwent fixation with 4% paraformaldehyde, paraffin-embedding, rehydration, and longitudinal sectioning into 4μm thick sections. The immunohistochemistry was performed to detect rat bFGF in sections. The specimens were stained overnight with mouse anti-rat bFGF (05-118, Millipore Corp., Billerica, Mass.), mouse anti-chicken PCNA antibody (ab29, Abcam, Cambridge, Mass.) at 1:3000 dilution in a humid chamber at 4 °C. The immunofluorescence was performed to examine tenocyte proliferation. For the sections with PCNA staining, the localizations of the PCNA protein were then visualized by incubating with fluorescein isothiocyanate–conjugated goat anti-mouse immunoglobulin G (ICL, Inc, Newberg, Oregon) at 1:200 dilution.

### *In situ* TUNEL Assay

Detection of cell death in the histological tissue section was done by TUNEL assay kit (Roche, Mannheim, Germany) according to the manufacturer’s protocol. Paraffin-embedded tissues were sectioned and incubated with TUNEL reaction mixture for 1 hour at 37 °C in a humidified chamber. Converter-Peroxidase (POD) solution was applied and the slides were incubated. The slides were incubated at ambient temperature after addition of the chromogenic substrate 3,3-diaminobenzidine (DAB), and were counterstained with Mayer’s hematoxylin.

### Western blot

The tendon samples were homogenized. Protein content was normalized and the samples were subjected to SDS-polyacrylamide gel electrophoresis and transferred onto a polyvinylidene difluoride membrane filter (Millipore Corp., Billerica, Mass.). The filters were incubated in phosphate-buffered saline containing 0.5% Tween 20 and 5% nonfat milk and then incubated with primary antibody overnight at 4 °C. After incubation with conjugated affinity-purified secondary antibody labeled with IRDye 800, blots were washed and immunoreactive proteins were scanned on an Odyssey imager (LI-COR, Inc., Lincoln, NE). Optical density on the membrane was measured and the relative differences between an internal control (ß-actin) and treated samples were calculated. Mouse anti-rat bFGF (Milipore Corp., Billerica, Mass.), mouse anti-human VEGF (Santa Cruz, Dallas, Texas), mouse anti-chicken MMP2 and TIMP2 (Abcam, Cambridge, Mass.) and mouse anti-chicken type I collagen and type III collagen (Acris, San Diego, Calif.) were used respectively as primary antibodies to detect different proteins.

### Quantification scoring of adhesion tissue of the tendons

An established grading method^19^ was used for grading adhesions macroscopically. With use of software (Reconstruct, Version 1.1.0.0; John C. Fiala, Boston, MA) for three-dimensional (3-D) reconstruction and alignment of serially sectioned samples of the tendons, we could verify the extent of adhesions recorded in the samples. We reconstructed the adhesions with tendons over a length of 1 cm. We applied the same 3-D reconstruction methodology to align sections stained with *in situ* TUNEL assay to examine the differences between apoptotic cells in the tendon surface and core.

### Biomechanical test of the healing strength

We harvested the FDP tendon through its entire length for the test of tendon strength in an Instron tensile testing machine (model 4411; Instron Inc., Norwood, Mass.). The distal phalanx attached with the terminal FDP tendon was mounted in the lower clamp of the machine. The proximal tendon end was mounted in the upper clamp. The length of the tendon was 8 cm between the two clamps with the repair site was maintained at the middle. The tendon was distracted linearly at a constant speed of 25 mm/min. The load on the tendons was continuously measured until ultimate failure, which was indicated by a sharp decline in load displacement shown on the monitor and abrupt disruption at the repair site. The forces were measured to the nearest 0.1N.

### Biomechanical test of resistance to the tendon: work of flexion and gliding excursion

The toes for quantifying resistance to toe motion were harvested through amputation at the knee joint and were mounted on a platform attached to the lower clamp of the testing machine (Instron). The proximal tendon was connected to the upper clamp. Both tendon gliding and work of toe flexion indicate resistance to digital motion, as mechanical measures of severity of adhesion formation^60^,^61^,^62^. With this setup, we measured (1) FDP tendon excursion under a fixed load (10N), and (2) the work of toe flexion, i.e., the energy required to flex the toe over a fixed for 70-degree from full extension. In testing the excursion, all toe joints were unrestricted, and tendon excursion was tested during the first run and work of flexion at the second run.

### Quantification and Statistics

Data are expressed as mean ± SD. In performing western blot analysis, we measured the density of target and control bands with a computer-assisted imaging analysis system. We counted the number of PCNA positively stained cells under fluorescence microscope. Ultimate tendon strength and gliding excursion were obtained from direct readout of the monitor. The load-displacement graph was recorded by the testing machine and energy required for digital flexion is work of flexion. Differences in gene expression, protein amount, and number of positively stained cells after PCNA or TUNEL staining, adhesion scores, tendon strengths, work of flexion, and tendon excursions were analyzed with two-way repeated measure of analysis of variance. A Tukey’s HSD test with Holm–Bonferroni correction was used as a *post hoc* test to detect significance between each pair of data comparisons. The criterion for statistical significance was P < 0.05.

## Additional Information

**How to cite this article**: Tang, J. B. *et al*. Basic FGF or VEGF gene therapy corrects insufficiency in the intrinsic healing capacity of tendons. *Sci. Rep.* **6**, 20643; doi: 10.1038/srep20643 (2016).
